# HIF-1α regulates COXIV subunits, a potential mechanism of self-protective response to microwave induced mitochondrial damages in neurons

**DOI:** 10.1038/s41598-018-28427-5

**Published:** 2018-07-10

**Authors:** Yan-Hui Hao, Jing Zhang, Hui Wang, Hao-Yu Wang, Ji Dong, Xin-Ping Xu, Bin-Wei Yao, Li-Feng Wang, Hong-Mei Zhou, Li Zhao, Rui-Yun Peng

**Affiliations:** 1 Department of Experimental Pathology, Beijing Institute of Radiation Medicine, Beijing, 100850 P.R. China; 2 Department of Radiation Protection and Health Physics, Beijing Institute of Radiation Medicine, Beijing, 100850 P.R. China

## Abstract

Anxiety and speculation about potential health hazards of microwaves exposure are spreading in the past decades. Hypoxia-inducible factor-1α (HIF-1α), which can be activated by reactive oxygen species (ROS), played pivotal roles in protective responses against microwave in neuron-like cells. In this study, we established 30 mW/cm^2^ microwave exposed animal model, which could result in revisable injuries of neuronal mitochondria, including ultrastructure and functions, such as ROS generation and cytochrome c oxidase (COX) activity. We found that the ratio of COXIV-1/COXIV-2, two isoforms of COXIV, decreased at 1 d and increased from 3 d to 14 d. Similar expression changes of HIF-1α suggested that COXIV-1 and COXIV-2 might be regulated by HIF-1α. In neuron-like cells, 30 mW/cm^2^ microwave down-regulated COX activity from 30 min to 6 h, and then started to recover. And, both HIF-1α transcriptional activity and COXIV-1/COXIV-2 ratio were up-regulated at 6 h and 9 h after exposure. Moreover, HIF-1α inhibition down-regulated COXIV-1 expression, promoted ROS generation, impaired mitochondrial membrane potentials (MMP), as well as abolished microwave induced ATP production. In conclusion, microwave induced mitochondrial ROS production activated HIF-1α and regulated COXIV-1 expression to restore mitochondria functions. Therefore, HIF-1α might be a potential target to impair microwave induced injuries.

## Introduction

Mitochondria play pivotal roles in maintaining energy metabolism, and mitochondrial dysfunctions are always associated with neurological damages, including non-infectious stimuli induced injuries^1–3^. Studies from our group and others demonstrated that microwave exposure, a type of electromagnetic radiation, could induce mitochondrial function disorder and mitochondrial structural injuries^4–7^. Interestingly, hypoxia-inducible factor-1α (HIF-1α), a key physiological sensor of oxygen level in most mammalian cells, was up-regulated in PC12 derived neuron-like cells after 30 mW/cm^2^ microwave exposure. Importantly, increased HIF-1α expression initiated the protective responses against microwave^5^. Under condition of non-infectious stimuli, such as hypoxia, ionizing and electromagnetic radiation, HIF-1α always acts as the master switch between acute response and adaptive response^8–10^. Recently, studies highlighted that mitochondrial disorder, such as increased ROS production, could directly or indirectly activate HIF-1α signaling in mammalian cells^11,12^. Importantly, numerous HIF-1α target genes, including pyruvate dehydrogenase kinase 1, BNIP3, lactate dehydrogenase A and complex IV, are critical in maintaining mitochondrial functions and energy metabolism^13,14^.

Cytochrome c oxidase (COX) subunits, complex IV in the mitochondrial respiratory chain, are located in the inner mitochondrial membrane and play pivotal roles in oxidative phosphorylation and ATP production^15^. COXIV, an important regulatory subunit of COX, could allosterically inhibit COX activity at high ATP/ADP ratios, through binding to ATP^16^. COXIV have two isoforms, COXIV-1 and COXIV-2. It has been reported that HIF-1α regulates COXIV subunit expression by activating transcription of the genes encoding COXIV-2 and LON, which is a mitochondrial protease for COXIV-1 degradation. Moreover, mammalian cells could optimize the efficiency of respiration, via altering COX subunit composition, to adapt hypoxia environment^17^. However, whether HIF-1α regulated COXIV subunits to protect neurons from microwave induced damages are still unknown.

In this study, we detected the HIF-1α, COXIV-1 and COXIV-2 protein expression both in the rat hippocampus and PC12 derived neuron-like cells. Moreover, we investigated the roles of HIF-1α in regulating COXIV subunits and in protecting mitochondrial functions in neuron-like cells *in vitro*.

## Materials and Methods

### Animals and microwave exposure

All the animal protocols were approved by the Animal Care and Use Committee at Beijing Institute of Radiation Medicine. And, all methods were performed in accordance with the relevant guidelines and regulations. Seventy-two male Wistar rats (203.7 g ± 8.87 g) were randomly divided into two groups: microwave exposure (MW) group and sham exposure (SH) group.

Pulsed microwaves at S-band with the frequency of 2.856 GHz, was generated by a klystron amplifier (model JD 2000) (Vacuum electronics research institute, Beijing, China). Microwave energy was transmitted by rectangular waveguide and A16-dB standard-gain horn antenna to an electromagnetic shield chamber (7 m × 6.5 m × 4 m). And, the diagonal of the antenna was 33 cm. To minimize reflections, interior walls of the chamber were covered with 500 mm and 300 mm pyramidal microwave absorber. The exposure table was 1.4 m below the lower edge of horn antenna, and the center of the table was just under middle of horn antenna. The animals were placed in a plastic cage inside a Plexiglas cylinder, which has a rotational symmetry around the center and contains twenty radial boxes for placing the rats. Microwave from the horn antenna irradiates animals from up to down (Supplementary Fig. 1). The peak power density was 200 W/cm^2^ in this study. And, the microwave pulse was delivered at 300 pps, with a pulse width of 500 ns. The GX12M30A power heads were fixed at one port of the circulator (plexiglas cylinder), and connected to GX12M1CHP power meter (Guanghua microelectronics instruments, Hefei, China) via waveguide antenna. The average power density was calculated to be 30 mW/cm^2^. And, the rats were exposed to microwave for at 30 mW/cm^2^ for 15 min^18–20^. During microwave exigposure, no significant environmental temperature elevation was detected. Moreover, the body temperature before and after exposure was measured by infrared imager (ThermaVision A40, FLIR systems, Wilsonville, OR, USA), and no obvious elevation was observed. For SH group, animals were processed in parallel to that in MW group, but without microwave exposure.

### Cell culture and microwave exposure

Rat pheochromocytoma (PC12) cells, a neuron precursor cell line, were cultured in Dulbecco’s Modified Eagle Medium (DMEM) (Gibco, Grand Island, NY) supplemented with 10% horse serum (Gibco) and 5% fetal bovine serum (FBS, Kang Yuan Biology, Tianjin, China). Neuron-like cells were induced by 5 ng/mL nerve growth factor (NGF, Sigma, St. Louis, MO) as described previously^5^. For exposure, neuron-like cells in 6-well plate were plated at the exposure table, and the average power density was monitored by GX12M30A power heads and GX12M1CHP power meter as described above. The cells in MW group were exposed to 30 mW/cm² microwaves for 6 min. Cells in SH group were treated as the MW group, but without microwave exposure.

### Transmission Electron Microscopy (TEM)

At 6 h, 7 d and 14 d after 30 mW/cm^2^ microwave exposure, hippocampus were dissected from the CA3 region under anatomical microscope. Then, the samples were fixed in 2.5% glutaraldehyde, sequentially processed with 1% osmium tetroxide, graded ethyl alcohols, and embedded in EPON618. Samples cut into ultrathin (70 nm) sections, and were stained with the heavy metals, uranyl acetate, and lead citrate for contrast. After drying, the ultrastructure of mitochondria was observed by TEM (HITACHI Ltd, Tokyo, Japan).

### Mitochondrial function assay

#### COX activity

At 6 h, 1 d, 3 d, 7 d, 14 d and 28 d after 30 mW/cm^2^ microwave exposure, rat hippocampus were collected, and then mitochondria were isolated by using Mitochondrial Isolation Kit (GENMED Scientifics INC., South San Francisco, CA). Mitochondria suspensions were collected. Then, COX activity was quantified by using COX Assay Kit (GENMED Scientifics INC., South San Francisco, CA) according to the manufacture’s instruction, and normalized by protein concentration.

#### ATP content

PC12 derived neuron-like cells were treated by the inhibitors of HIF-1α (Bisphenol A) at 1 h before microwave exposure. At 9 h after exposure, cells were collected and schizolysed, and then the supernatant were collected. The ATP content was measured by using a Firefly Luciferase-based ATP Kit (Beyotime, Nanjing, China) as described previously^5^, and normalized by protein content.

#### Mitochondrial Membrane Potentials (MMP)

At 9 h after microwave exposure, inhibitors treated neuron-like cells and control cells were used for detecting MMP by using a JC-1 MMP Assay Kit (Beyotime, Nanjing, China). Briefly, cells in 6-well plates were washed with PBS and incubated with JC-1 staining solution at 37 °C for 20 min. After rinsed twice with JC-1 staining buffer, the MMPs were monitored by fluorescent microscope (Olympus, Tokyo, Japan) and were quantified by flow cytometry (BD Bioscience, San Jose, CA). And, relative MMP were calculated.

#### Intracellular ROS Production

At 9 h after microwave exposure, inhibitors treated neuron-like cells and control cells were collected and incubated with 2′,7′-dichlorofluorescein diacetate (DCFH-DA) (Beyotime, Nanjing, China) at 37 °C for 20 min. Then, fluorescent DCF were measured by flow cytometry to evaluate the ROS level using. Moreover, at appointed time points after exposure, the hippocampal mitochondria were isolated to detect the ROS content using ROS Detection Kit (GENMED Scientifics INC., South San Francisco, CA). And, ROS content was normalized by mitochondrial concentration.

### Immunohistochemistry

Rat brains were removed at 6 h, 3 d, 7 d, 14 d and 28 d after microwave exposure, and were fixed in 10% buffered formalin solution, embedded in paraffin and cut at 3 μm thick in the coronal plane. The HIF-1α protein was detected by immunohistochemistry using rabbit anti-rat HIF-1α polyclonal antibody (Bioworld Technology, MN). And, HIF-1α expression was observed blindly under a light microscope (Leica, Wetzlar, Germany). The HIF-1α expressed cells were filled with brown and yellow granules. Ten random visions were selected, and the rate of HIF-1α expressed cells to total cells was calculated.

### Immunofluorescence staining

At 9 h after microwave exposure, neuron-like cells were labeled with rabbit anti-rat HIF-1α polyclonal antibody overnight at 4 °C, and then incubated with FITC conjugated goat anti-rabbit IgG secondary antibody (ZSGB-BIO, Beijing, China) for 1 h at room temperature. The HIF-1α expression was observed by fluorescent microscope after DAPI staining.

### HIF-1α inhibition

To investigate the roles of HIF-1α in the protective responses against microwave, we blocked HIF-1α protein expression by adding HIF-1α inhibitor Bisphenol A (400 μM) to neuron-like cells at 1 h before exposure. And, at 9 h after exposure, the HIF-1α, COXIV-1 and COXIV-2 expression, mitochondrial functions, such as ATP, MMP and ROS, were evaluated.

### Western blot

Total protein and nuclear protein (Nuclear Protein Extraction kit, BestBio, Shanghai, China) were extracted from rat hippocampus, neuron-like cells, Bisphenol A treated neuron-like cells at appointed time points after microwave exposure. Protein expression was detected by rabbit anti-rat HIF-1α antibody (Bioworld Technology, MN), mouse anti-rat COXIV-1 antibody (Thermo Fisher Scientific, Rockford, IL), goat anti-rat COXIV-2 antibody (Santa Cruz Biotechnology, Dallas, TX), rabbit anti-Lamin B1 antibody (Bioworld Technology, MN) and anti-glycolytic glyceraldehyde-3-phosphate dehydrogenase (GAPDH) antibody (KangChen Biology, Nanjing, China). The protein expression was normalized by GAPDH or Lamin B.

### Real-time reverse transcript polymerase chain reaction (RT-PCR)

Total RNA was isolated from hippocampus after microwave exposure. Then, the complementary DNA (cDNA) was synthesized by using RevertAid First Strand cDNA Synthesis Kit (Thermo Scientific, Wilmington, DE). The mRNA expression of HIF-1α was measured by real-time RT-PCR using Power SYBR@ Green PCR Master Mix (Thermo Fisher Scientific, Wilmington, DE), and normalized by the expression of GAPDH. The specific primers were shown as followed, HIF-1α forward, 5′- tctagtgaacaggatggaatggag-3′ and reverse, 5′- tcgtaactggtcagctgtggtaa-3′; GAPDH forward, 5′- ggcacagtcaaggctgagaatg-3′ and reverse 5′- atggtggtgaagacgccagta-3′.

### Statistical analysis

Data were presented as mean ± s.e.m. and statistically analyzed by using GraphPad Prism software version 5 (GraphPad software, San Diego, CA). One-way ANOVA followed by Bonferroni post hoc tests were performed to analyze multiple groups. Longitudinal data were analyzed by using a two-way repeated measure ANOVA followed by Bonferroni post hoc tests for all the data of over time course. Differences were considered significant at two sided p < 0.05.

## Results

### Microwave damages ultrastructure of mitochondria, increases ROS production and impairs COX activity in rat hippocampus

**Figure 1:**
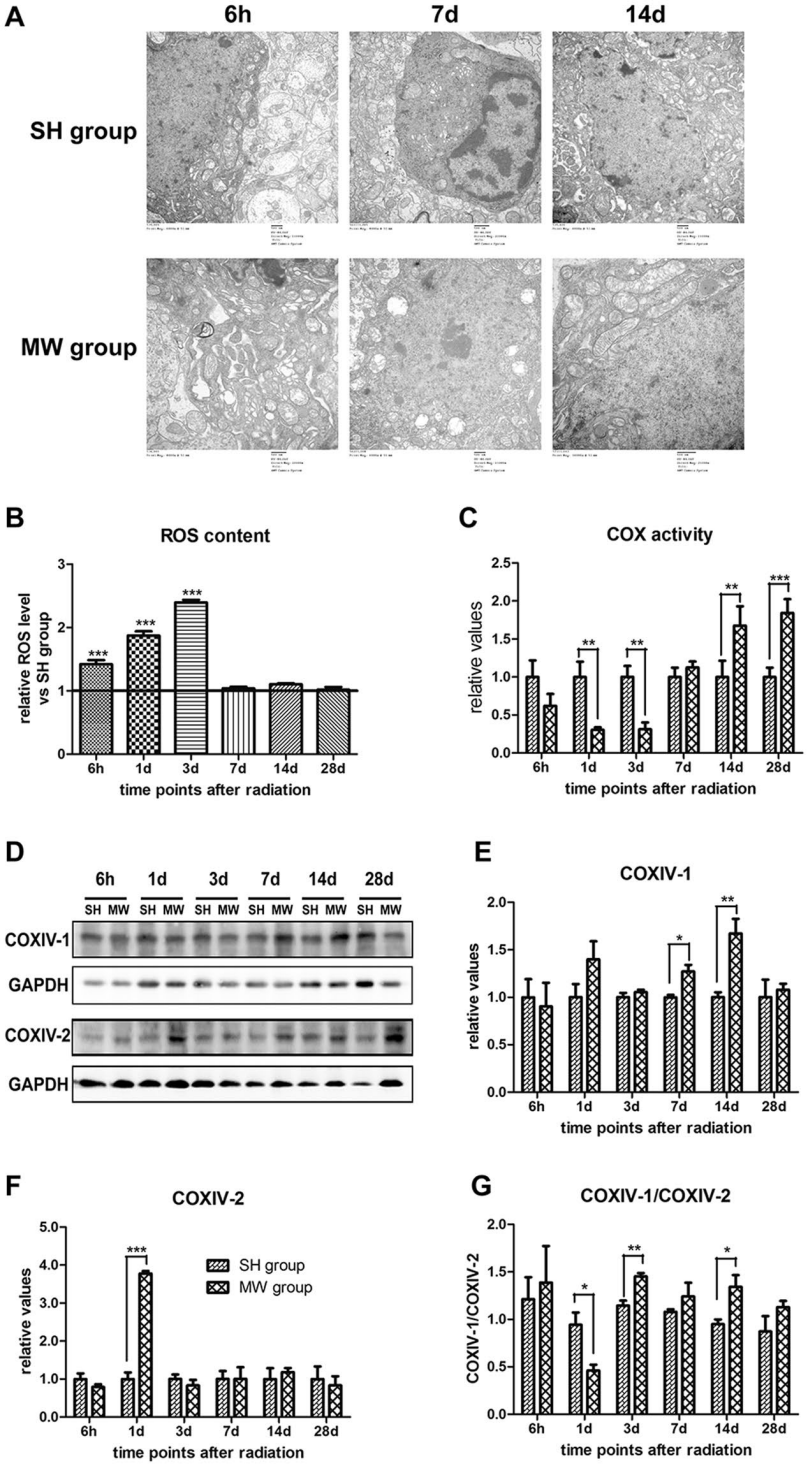
Mitochondrial ultrastructure, reactive oxygen species generation, activity of cytochrome c oxidase (COX), and dynamic expression of COXIV subunits in rat hippocampal tissues after microwave exposure. Seventy-two rats were exposed to 30 mW/cm^2^ microwave for 15 min. (**A**) Ultrastructure of mitochondria in hippocampus. After exposure, 5 rats from each group were euthanized and the hippocampus were isolated at appointed time points. The ultrastructure of mitochondria was observed by transmission electron microscopy after staining with heavy metals. And, the representative images were shown. (**B**) Mitochondrial ROS Production. Mitochondria were isolated from hippocampus. The ROS content in mitochondrial suspension was detected via calculating the conversion from DCFH-DA to DCF, and normalized by mitochondrial concentration. The relative ROS production to that in SH group was presented. (**C**) COX activity. The COX activity in mitochondrial suspension was quantified by using COX assay kit. (**D**–**F**) Dynamic expression of COXIV subunits. After microwave exposure, hippocampus was isolated. The COXIV-1 and COXIV-2 protein expression was analyzed by western-blotting. The representative images were shown in D, and quantitative analysis was presented in E and F. Moreover, the ratio of COXIV-1 to COXIV-2 was also presented in G. Data were shown as mean ± s.e.m. **p < 0.01, ***p < 0.001 vs corresponding group.

We have previously reported that 30 mW/cm^2^ microwave induced rat hippocampal injuries^18,21–23^. Mitochondria are the pivotal organelle to maintain energy metabolism. Therefore, we observed the ultrastructure of mitochondria in rat hippocampus after microwave exposure. In SH group, round or oval mitochondria were well-distributed in size and shape, with clear boundary and intact mitochondrial cristae. After microwave exposure, slight disorder of mitochondrial cristae could be observed at 6 h, while aggravated injuries, such as swelling, cavitation, and broken or disappeared mitochondrial cristae were observed at 7 d. Interestingly, mitochondria started to recover at 14 d after exposure and showed alleviated injuries (Fig. 1A).

Mitochondrial respiratory chain plays critical roles in mitochondrial respiration and energy metabolism. We showed that intracellular ROS was up-regulated significantly from 6 h to 3 d after 30 mW/cm^2^ microwave exposure (Fig. 1B). Meanwhile, microwave significantly reduced the activity of COX at 1 d and 3 d, while increased the activity at 14 d and 28 d after exposure (Fig. 1C). These results indicated that microwave could regulate the efficiency of mitochondrial respiratory chain.

COXIV contains two different isoforms: COXIV-1 and COXIV-2, and the balance between COXIV-1 and COXIV-2 plays important roles in maintaining oxidation phosphorylation. We showed that microwave increased COXIV-1 protein at 7 d and 14 d, while up-regulated COXIV-2 expression at 1 d after exposure. Moreover, the ratio of COXIV-1/2 decreased at 1 d and increased from 3 d to 14 d (Fig. 1D–G). These results suggested that COXIV-1 and COXIV-2 participated in the mitochondrial injuries and recovery after microwave exposure.

### Microwave induces dynamic expression of HIF-1α in rat hippocampus

**Figure 2:**
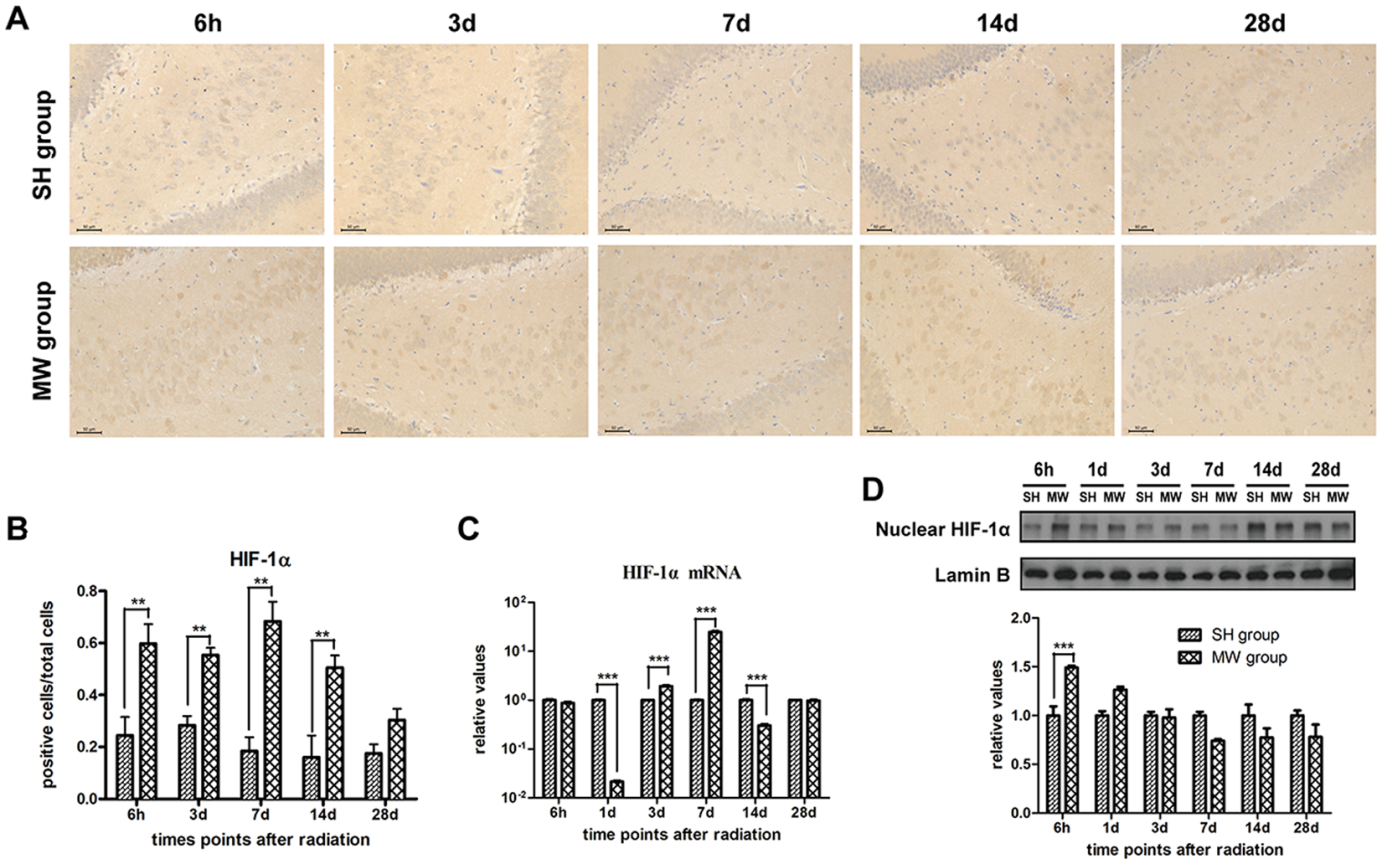
Dynamic expression of hypoxia-inducible factor-1α (HIF-1α) in rat hippocampal tissues after microwave exposure. (**A**,**B**) HIF-1α protein expression. Rat brains were removed after microwave exposure at various time points, and the HIF-1α protein expression *in situ* was detected by using immunohistochemistry. The representative images were shown in A, and the statistical analysis were presented in B. (**C**) HIF-1α mRNA expression. Hippocampus was isolated from microwave exposed rats. Then, the total RNA was extracted and cDNA was synthesized. HIF-1α mRNA expression was detected by real-time reverse transcript polymerase chain reaction, and normalized by GAPDH expression. (**D**) Nuclear HIF-1α protein. The nuclear protein from rat hippocampus was prepared, and the HIF-1α protein expression was analyzed by western-blotting and normalized by Lamin B. Data were shown as mean ± s.e.m. ***p < 0.001 compared to the corresponding group.

Our previous work has demonstrated that HIF-1α could protect neuron-like cells from microwave-induced mitochondrial disorder. In this study, we showed that HIF-1α protein expression was up-regulated in rat hippocampus from 6 h to 14 d after microwave exposure (Fig. 2A,B). Similar HIF-1α mRNA expression was detected in microwave exposed rat hippocampus (Fig. 2C). Moreover, nuclear HIF-1α level was increased both at 6 h and 1 d after microwave exposure (Fig. 2D). These results suggested that HIF-1α could activate its transcriptional activities to protected neurons from microwave induced mitochondrial injuries.

### Microwave regulates COXIV-1 and COXIV-2 expression, and COX activity in neuron-like cells

**Figure 3:**
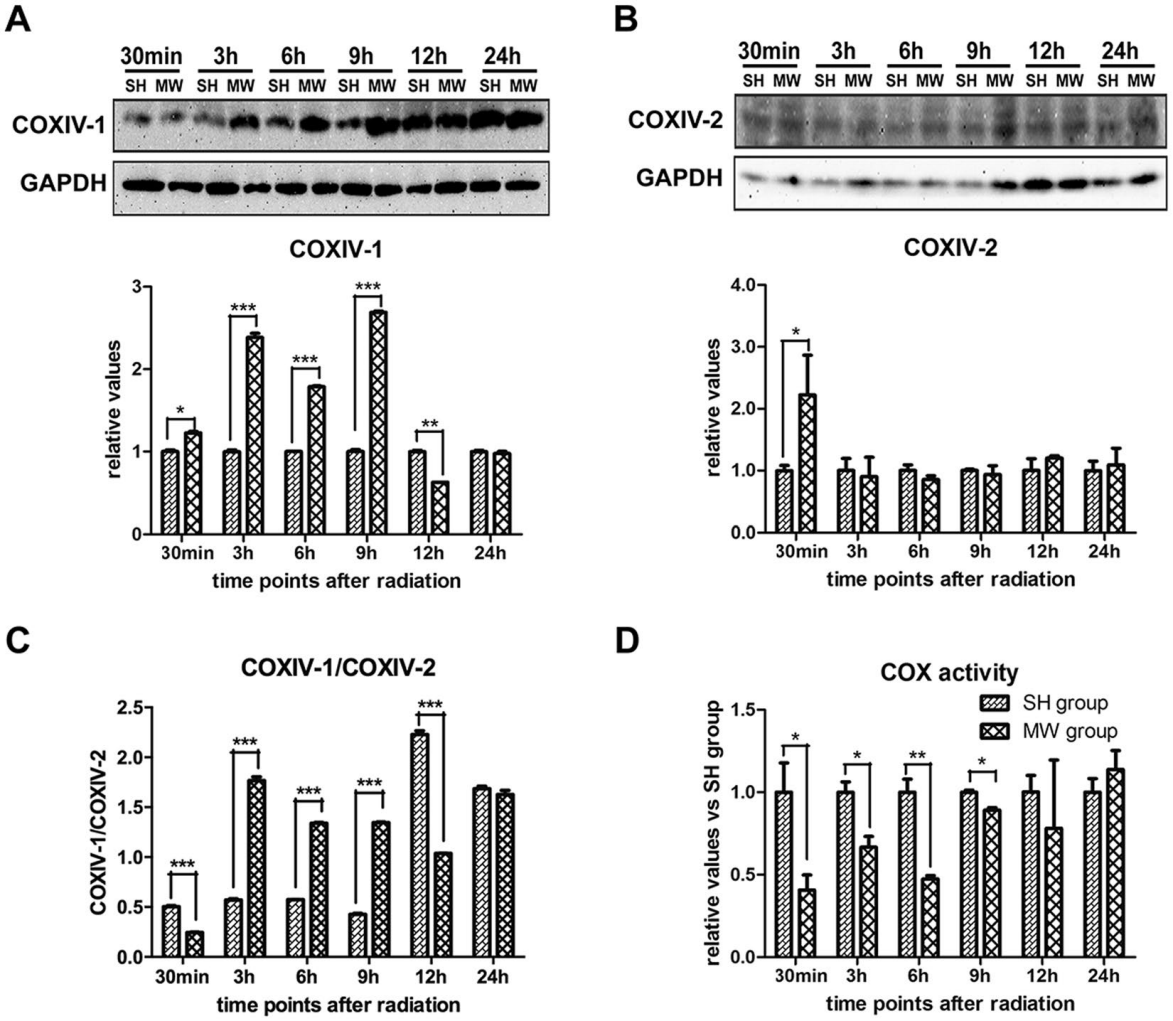
COXIV-1 and COXIV-2 expression, and COX activity in PC12 cell derived neuron-like cells. Neuron-like cells were induced from rat pheochromocytoma (PC12) cells, by 5 ng/mL nerve growth factor. Then, neuron-like cells were exposed to 30 mW/cm² microwaves for 6 min. Cells were harvested at various time points after exposure. Protein expression of COXIV-1 (**A**) and COXIV-2 (**B**) were analyzed by western-blotting. And, the ratio of COXIV-1 to COXIV-2 was presented in (**C**). Moreover, The COX activity was detected by COX assay kit and normalized by protein concentration (**D**). Data were shown as mean ± s.e.m. *p < 0.05, **p < 0.01 compared to corresponding group.

To investigate the mechanisms under HIF-1α mediated protective responses, we established an *in vitro* model in PC12-derived neuron-like cells. In this model, COXIV-1 was up-regulated from 30 min to 9 h and down-regulated at 12 h after exposure, while COXIV-2 was up-regulated at 30 min after exposure (Fig. 3A,B). Moreover, the ratio of COXIV-1/2 decreased at 30 min, and increased from 3 h to 9 h after exposure (Fig. 3C). Meanwhile, COX activity significantly decreased from 30 min to 9 h, and then restored to normal level at 12 h after exposure (Fig. 3D). These results suggested that increasing COXIV1/2 might drive the recovery of mitochondrial functions.

### Microwave exposure up-regulates the transcription activity of HIF-1α in neuron-like cells

**Figure 4:**
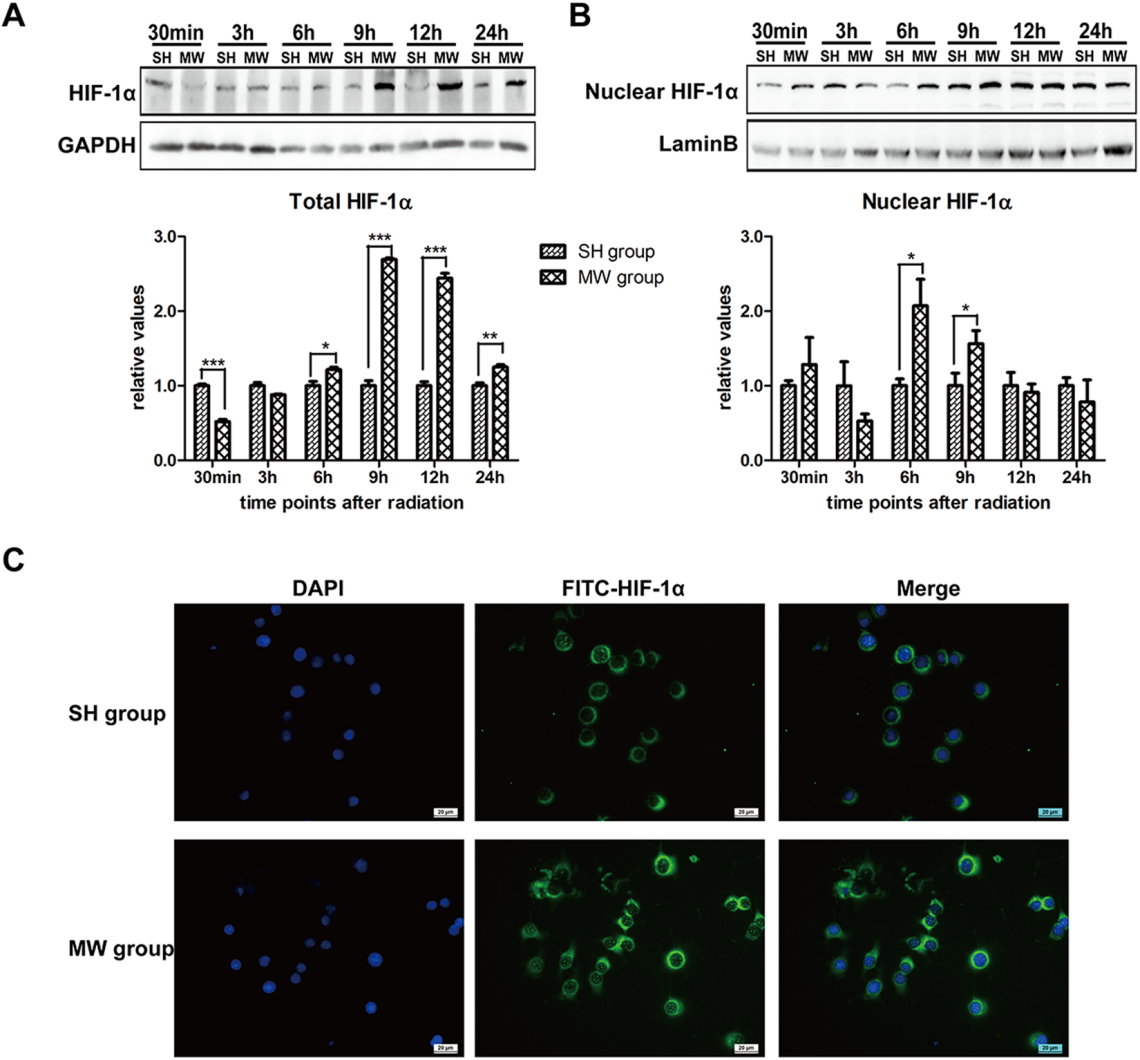
Microwave exposure promotes HIF-1α nuclear translocation in neuron-like cells. Neuron-like cells were exposed to 30 mW/cm^2^ microwave. After exposure, the total HIF-1α protein expression was detected by western-blotting, and normalized by GAPDH (**A**). Moreover, nuclear protein was also extracted. Then, nuclear HIF-1α level was detected by western-blotting, and normalized by Lamin B (**B**). Immunofluorescence staining was conducted to show the expression and distribution of HIF-1α. The representative images were captured under fluorescent microscope (**C**). Data were shown as mean ± s.e.m. *p < 0.05, **p < 0.01 vs corresponding group.

Then, we showed that microwave induced HIF-1α expression from 9 h to 24 h after microwave exposure (Fig. 4A). Further analysis suggested that microwave increased the level of nuclear HIF-1α, especially at 6 h and 9 h after exposure, indicating activation of HIF-1α mediated transcription (Fig. 4B). The increase of nuclear HIF-1α level was also confirmed by immunofluorescence. These results suggested that microwave could increase HIF-1α expression and enhance its transcriptional activity.

### HIF-1α regulates COXIV-1 and COXIV-2 expression to protect mitochondrial functions after microwave

**Figure 5:**
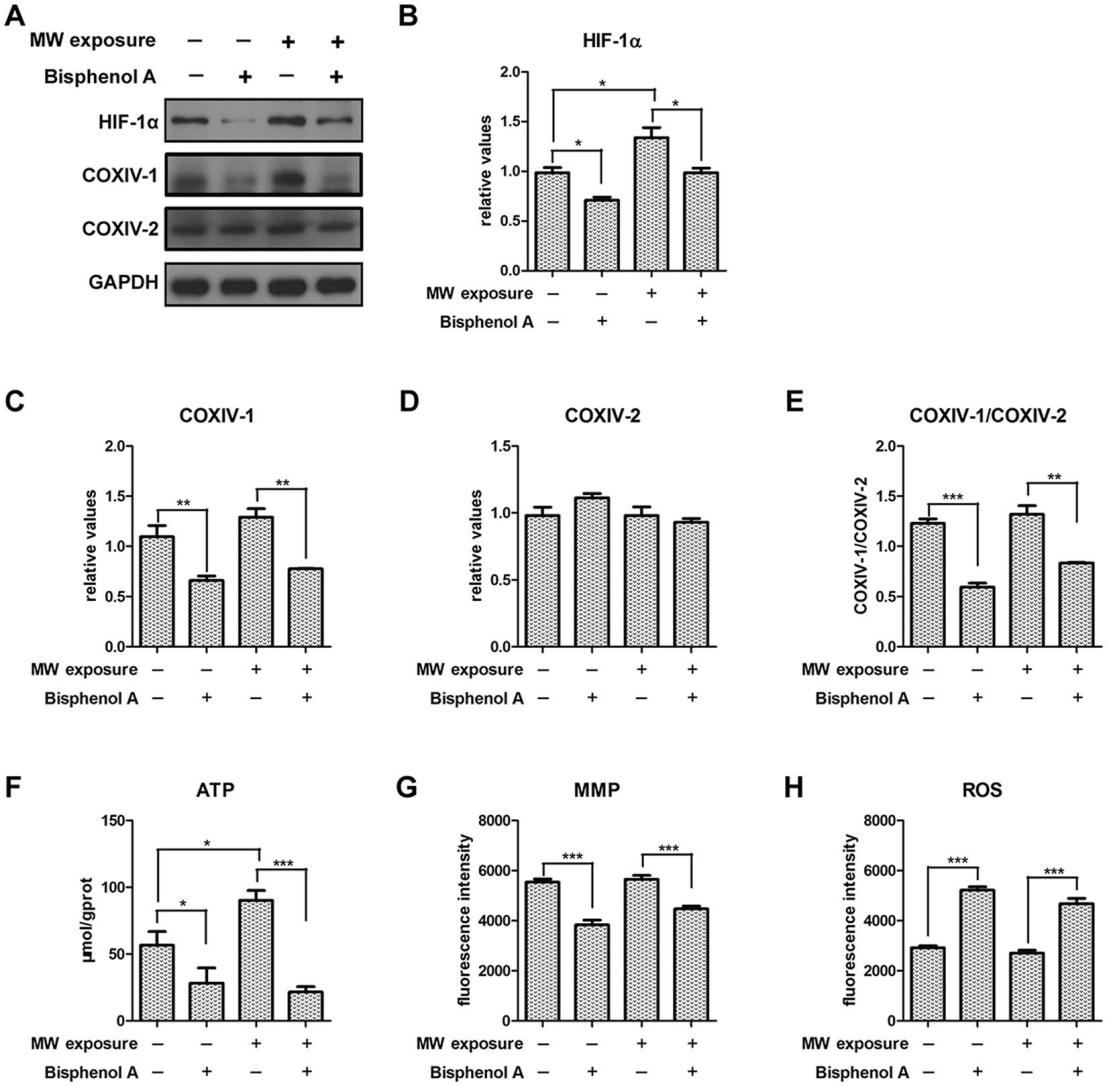
HIF-1α inhibition abolishes the up-regulation of COXIV-1 after microwave exposure and impairs mitochondrial functions in neuron-like cells. Neuron-like cells were treated with 400 μM Bisphenol A at 1 h before microwave exposure. At 9 h after exposure, cells were collected. The protein expression of HIF-1α, COXIV-1 and COXIV-2 were detected by western-blotting (**A**). And, the quantitative analysis were presented in (**B**), (**C**) and (**D**), respectively. The ratio of COXIV-1 to COXIV-2 was also calculated (**E**). To evaluate the mitochondrial functions, the ATP content (**F**), the mitochondrial membrane potentials (MMP) (**G**), and mitochondrial ROS (**H**) was analyzed by commercial kit. Data were shown as mean ± s.e.m. *p < 0.05, **p < 0.01, ***p < 0.001 compared to corresponding group.

HIF-1α protein was inhibited after treated with Bisphenol A, at 9 h after microwave exposure (Fig. 5A,B). HIF-1α inhibition reduced COXIV-1 protein, but had no obvious effects on COXIV-2. Moreover, the ratio of COXIV-1 to COXIV-2 was down-regulated after inhibiting HIF-1α (Fig. 5A,C–E). These results suggested that COXIV-1 expression was regulated by HIF-1α in neuron-like cells.

Then, we analyzed mitochondrial functions, such as ATP level and MMP. Microwave elevated ATP level at 9 h after microwave exposure, suggesting recovery mechanism has been initiated. However, HIF-1α inhibition not only reduced ATP production in sham-exposed cells, but also abolished microwave induced up-regulation of ATP (Fig. 5F). Moreover, HIF-1α inhibitor also down-regulated MMP (Fig. 5G). Dysfunction of mitochondrial respiratory chain could produce ROS to damage structures and functions of corresponding tissues and organs. We found HIF-1α inhibition could increase ROS level, in both sham- and microwave-exposed cells (Fig. 5H). Therefore, our results indicated that HIF-1α played pivotal roles in protecting mitochondrial functions after microwave exposure, via regulating COXIV-1 expression and COX activity.

## Discussion

The anxiety and speculation about potential health hazards caused by microwave radiation, nonionizing electromagnetic radiation ranging from 300 MHz to 300 GHz, has been growing fast in recent years^24^. Our group and others have been focusing on the biological effects of microwave exposure and its mechanisms^5,18,21–23,25–28^. Our previous work revealed the pivotal roles of HIF-1α in the self-protective response to 30 mW/cm^2^ microwave in neuron-like cells. Mitochondria are the major sites of ROS production, which could cause oxidative damages. Importantly, HIF-1α mediated self-protection could be activated by increased ROS^11,12,29,30^. In this study, we investigated the mechanisms under HIF-1α mediated protective responses against microwave. We showed that HIF-1α could be activated both in rat hippocampus and neuron-like cells. And, HIF-1α inhibition could down-regulate the ATP production and decrease MMP of mitochondria, while increase intercellular ROS level.

Under hypoxia or other non-infectious stimuli conditions, mitochondrial ROS can prevent the hydroxylation of HIF-1α and stabilize HIF-1α. Then, stabilized HIF-1α proteins translocate to the nucleus and dimerize with HIF-1β subunit to form heterodimeric protein and activate transcription of numerous target genes, such as pyruvate dehydrogenase kinase 1, BNIP3, lactate dehydrogenase A and complex IV, miRNAs, and so on^8,10,31,32^. In this study, we demonstrated that microwave could up-regulated nuclear HIF-1α level, suggesting the activation of HIF-1α mediated transcription. We speculated that the activation of HIF-1α might be one of the mechanisms to promote recovery of COX activity in rat hippocampus after microwave exposure.

COXIV is a regulatory subunit for COX activity, and interacts with COXI and COXII^15^. In complex IV, COXIV binds ATP and acts as a sensitive master switch, which can result in allosteric inhibition of COX activity under high ATP/ADP ratio^16^. It has been reported that two isoforms of COXIV, COXIV-1 and COXIV-2 were O_2_ regulated in mammalian cells. In hypoxic mammalian cells, up-regulated HIF-1α can activate transcription of COXIV-2 and LON genes, encoding a protease for COXIV-1 degradation^17^. In this study, we found that dynamic expression of COXIV-1 and COXIV-2 protein could be observed after microwave exposure. Briefly, an obvious down-regulation of the COXIV-1/COXIV-2 ratio was detected at the early stage, and then rapidly restored. And, a significant increase could be induced at the later stage, in accordance with the HIF-1α expression. These results suggested that HIF-1α modulated COX activity through regulating the expression of COXIV-1 and COXIV-2. Previous studies have demonstrated that the switch from COXIV-1 to COXIV-2 represents an adaptive response that optimizes COX activity, O_2_ consumption, and ROS generation, which can reduce the oxidative damages of mitochondria under hypoxic conditions. Therefore, up-regulation of COXIV-2 at the early stage after exposure was a potential mechanism to protect neurons from mitochondrial oxidative damages.

In conclusion, microwave exposure promoted ROS production in mitochondria, which could dynamically regulate HIF-1α expression both in rat hippocampus and PC12 derived neuron-like cells. Then, HIF-1α regulated COXIV-1 and COXIV-2 expression to protect mitochondria from microwave induced injuries, including oxidative damages. The ratio of COXIV-1 to COXIV-2 were dynamically changed in animal model, which indicated that different mechanisms were involved in HIF-1α mediated protective responses at different stages after microwave exposure. Combining with previous reports^13,14,17,33^, we speculated that at early stage after microwave exposure, increased HIF-1α protein could switch the COXIV proteins from COXIV-1 to COXIV-2, which could optimize the efficiency of mitochondrial respiratory chain. At the later stage, increased HIF-1α participated in the up-regulation of COXIV-1 to enhance oxidative phosphorylation in mitochondria.

## Electronic supplementary material

Supplementary Figure 1 Schematic diagram of experimental setup for microwave exposure
