# Supplementary material for: HIF-1α regulates COXIV subunits, a potential mechanism of self-protective response to microwave induced mitochondrial damages in neurons
Source: Sci Rep. 2018 Jul 10;8:10403. doi: 10.1038/s41598-018-28427-5 (PMC6039499; doi:10.1038/s41598-018-28427-5)

**HIF-1α regulates COXIV subunits, a potential mechanism of self-protective response to microwave induced mitochondrial damages in neurons**

Yan-Hui Hao^1^, Jing Zhang^1^, Hui Wang^1^, Hao-Yu Wang^1^, Ji Dong^1^, Xin-Ping Xu^1^, Bin-Wei Yao^1^, Li-Feng Wang^1^, Hong-Mei Zhou^2^, Li Zhao^1*^ and Rui-Yun Peng^1*^

**Supplementary Figure 1** Schematic diagram of experimental setup for microwave exposure


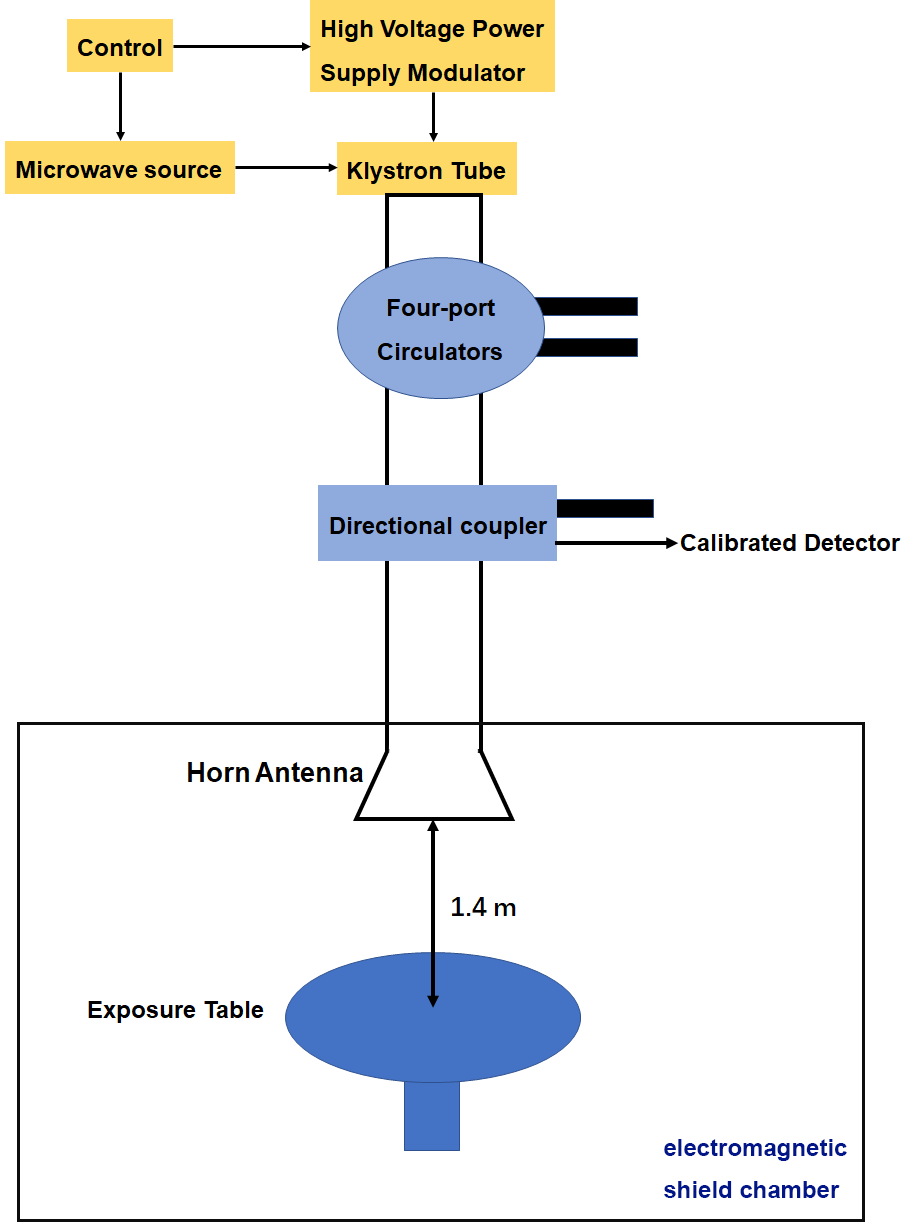

Supplement: Supplementary file 1 — Supplementary Figure 1 Schematic diagram of experimental setup for microwave exposure [file 41598_2018_28427_MOESM1_ESM.doc]
